# Antidiabetic, hypolipidemic and hepatoprotective effects of *Arctium lappa* root’s hydro-alcoholic extract on nicotinamide-streptozotocin induced type 2 model of diabetes in male mice

**Published:** 2017

**Authors:** Akram Ahangarpour, Hamid Heidari, Ali Akbar Oroojan, Farhang Mirzavandi, Khalil Nasr Esfehani, Zeinab Dehghan Mohammadi

**Affiliations:** 1 *Health Research Institute, Diabetes Research Center, Department of Physiology, Ahvaz Jundishapur University of Medical Sciences, Ahvaz, Iran *; 2 *Department of Physiology, Health Research Institute, Diabetes Research Center, Ahvaz Jundishapur University of Medical Sciences, Ahvaz, Iran. Department of Physiology, School of Medicine, Qom University of Medical Sciences, Qom, Iran *; 3 *Department of Physiology, Student Research Committee, Ahvaz Jundishapur University of Medical Sciences, Ahvaz, Iran *; 4 *Department of Nutrition, Student Research Committee, Ahvaz Jundishapur University of Medical Sciences, Ahvaz, Iran*

**Keywords:** Arctium Lappa, Type 2 diabetes, Insulin, Lipid profile, Hepatic enzyme

## Abstract

**Objective:**

*Arctium lappa* (burdock), *(A. lappa)* root has hypoglycemic and antioxidative effects, and has been used for treatment of diabetes in tradition medicine. This study was conducted to evaluate the antidiabetic and hypolipidemic properties of *A. lappa* root extract on nicotinamide-streptozotocin (NA-STZ)-induced type2 diabetes in mice.

**Materials and Methods:**

In this investigation, 70 adult male NMRI mice (30-35g) randomly divided into 7 groups (n=10) as follow: 1-control, 2-type 2 diabetic mice, 3-diabetic mice that received glibenclamide (0.25 mg/kg) as an anti-diabetic drug, 4, 5, 6 and 7- diabetic and normal animals that were pre-treated with 200 and 300 mg/kg *A. lappa* root extract, respectively, for 28 days. Diabetes has been induced by intraperitoneal injection of NA and STZ. Finally, the blood sample was taken and insulin, glucose, SGOT, SGPT, alkaline phosphatase, leptin and lipid levels was evaluated.

**Results:**

Induction of diabetes decreased the level of insulin, leptin and high density lipoprotein (HDL) and increased the level of other lipids, glucose, and hepatic enzymes significantly (p<0.05). Administration of both doses of the extract significantly decreased the level of triglyceride, very low density lipoprotein, glucose and alkaline phosphatase in diabetic mice (p<0.05). Insulin levels increased in animals treated with 200 mg/kg (p<0.05) and HDL and leptin levels increased in animals treated with 300 mg/kg of the extract (p<0.01).

**Conclusion:**

The results showed that *A. lappa* root extract, at specific doses, has an anti-diabetic effect through its hypolipidemic and insulinotropic properties. Hence, this plant extract may be beneficial in the treatment of diabetes.

## Introduction

Diabetes mellitus as a metabolic disorder was associated with various dysfunctions. High-calorie diet, abdominal obesity and sedentary lifestyle in aging population have increased the population of diabetic patients, worldwide (Horwich and Fonarow, 2010 ▶). Consistent with the World Health Organization (WHO) reports, the prevalence rate of diabetes mellitus will be 552 million and it will be among the top seven cause of death by the year 2030 (Whiting et al., 2011 ▶; Hadjzadeh et al., 2016 ▶). Reactive oxygen species (ROS) and oxidative stress result in apoptosis of beta cells and reducing insulin production by pancreatic islets. Therefore, oxidative stress has a critical role in increasing blood sugar, and pathogenesis and progression of diabetes mellitus (Yilmaz et al., 2013 ▶). Despite the presence of different types of chemical hypoglycemic agents such as biguanides and sulphonylureas for treatment of diabetes, due to the adverse effects and cost of these chemical agents, there is a growing tendency to take herbal medicine to control and treat this disorder (Chander et al., 2011 ▶). About 300 antidiabetic herbal medicines have been identified (Ahangarpour et al., 2014 ▶); one of them is *Arctium lappa* L. (*A. Lappa)* which belongs to the Asteraceae family and grows in humid temperate areas of Asia and Europe. This plant has fusiform brown roots that have been used as a traditional treatment for diabetes mellitus (Pandey et al., 2007 ▶). In Iranian traditional medicine, it has been noted that the root of this plant has blood sugar-lowering effect (Zargari, 1993 ▶). Anti inﬂammatory, hepatoprotective and free radical scavenging activities of *A. lappa* root have also been reported by several pharmacological studies and clinical trial investigations (Sohn et al., 2011 ▶; Lin et al., 2002 ▶). Phytochemical investigations indicated that *A. lappa* root is a rich source of phenols, saponins, lignans, tannin and flavonoids (Al-Shammaa et al., 2013 ▶). Flavonoids are polyphenolic components which reduce diabetes complications by scavenging free radicals (Song et al., 2005 ▶; Abbasnezhad et al., 2015 ▶). *A. lappa* root aqueous extract could induce hypoglycemia and hyperinsulinemia in our previous study in a sucrose model of diabetes (Ahangarpour et al., 2013 ▶). Furthermore, 14-day administration of *A. lappa* root ethanolic extract showed hypoglycemic and hypolipidemic effects on STZ induced type1 diabetes in rats (Cao et al., 2007 ▶). But, there is no study on type 2 diabetes focusing on prevention of total pancreas destruction by nicotinamide. So, the present study evaluated antidiabetic, hypolipidemic and hepatoprotective potentials of *A. lappa* root extract on nicotinamide-streptozotocin (NA-STZ)-induced type 2 model of diabetes in male mouse.

## Materials and Methods

**Plant material**

Fresh roots of *A.* *lappa* were obtained from the mountains of Isfahan province, Iran and scientifically validated by Department of the Botany of Ahvaz Jundishapur University of Medical Science, Ahvaz, Iran. A voucher specimen of *A.* *lappa* was deposited in the Herbarium of Faculty of Agriculture of Shahid Beheshti University (No. AR337E). The roots were crushed using mechanical grinders.

**Extract preparation**

To prepare hydro-alcoholic extract of *A.* *lappa* root, 50 g of its powder was macerated in 200 mL of mixture (60-40; distilled water-methanol) and kept for 72 hr at room temperature. Then, this mixture was filtered using Whatman filter papers (No. 1) and, centrifuged at 3500 rpm for 20 min. Eventually, the mixture was desiccated at room temperature, the solvent has been evaporated and, the acquired semisolid mass was kept at 4°C until used (Ahangarpour et al., 2014 ▶).

**Animals**

Seventy adult male NMRI mice (30-35 g) were purchased from animal house of Ahvaz Jundishapur University of Medical Sciences (AJUMS) and kept in cages at 20 ± 4°C temperature with 12 hr light /12 hr dark cycle and free access to tab tap water and commercial chow. All experimental protocols were in accordance with the guidelines and standards of animal’s care approved by the Institutional Animal Ethics Committee of AJUMS (Ahvaz, Iran) with ethical number D-9103.

**Type 2 model of diabetes mellitus** **induction**

This model of diabetes was induced in overnight-fasted adult male NMRI mice by a single intraperitoneal (IP) injection of NA (110 mg/kg body weight, dissolved in normal saline) (Merck, Germany) 15 min before IP administration of STZ (50 mg/kg body weight, dissolved in citrate buffer, pH 4.5) (Sigma Aldrich, USA) (Ahangarpour et al., 2015 ▶). The blood glucose level was measured before and 72 h after NA-STZ injection, for confirmation of hyperglycemia and type 2 diabetes development. Ultimately, the blood glucose levels above 250 mg/dL, were considered as diabetic and used for the experiments (Ahangarpour et al., 2014 ▶).

**Experimental design**

All mice were randomly divided into seven groups (10 mice per group) and, treated once a day for 28 sequential days in the following groups: control group which received normal saline; type 2 diabetes group; type 2 diabetes group treated with glibenclamide (0.25 mg/kg)(Sigma Aldrich, USA), as a standard hypoglycemic drug; two diabetes treatment groups which received *A. lappa* root hydro-alcoholic extract orally by gastric tube at the doses of 200 and 300 mg/kg body weight, respectively; and two groups of normal animals that received *A. lappa* root hydro-alcoholic extract 200 and 300 mg/kg orally by gastric tube respectively (Ahangarpour et al., 2015 ▶).

**Biochemical assessment**

One day after the last drug administration, the overnight-fasted animals were anesthetized by ether. Fasting blood glucose levels were assessed by an Elegance glucometer (CT-X10, Convergent Technologies, Germany) using the lateral tail vein of the mice on the first and last days of the experiment. Then, blood samples were directly collected by cardiac puncture and centrifuged at 3500 rpm for 20 min. Serum samples were kept at -70°C until biochemical assessment (Zamami et al., 2008 ▶). Serum insulin levels were evaluated by radioimmunoassay (RIA) (Diosource INS-IRMA Kit) with assay sensitivity of 1 µIU/mL, inter-assay coefficient of variation (CV) of 6.5% and intra-assay CV of 2.1%. Also, insulin resistance (HOMA-IR), homeostatic model assessment of pancreatic beta cell function (HOMA-β), quantitative insulin sensitivity check index (QUICKI) and insulin disposition index (DI) were calculated by the following formula: 

HOMA-IR: fasting blood glucose (mg/dL) × insulin (µIU/mL) / 405 (Ahangarpour et al., 2014 ▶).

HOMA-β: 20 × insulin (µIU/mL) / (FBS (mmol/L) - 3.5) (Ma et al., 2014 ▶)

QUICKI: 1 / (log FBS (mg/dL) + log insulin (µIU/mL)) (Ma et al., 2014 ▶)

DI: Ln HOMA-β /Ln HOMA-IR (Li et al., 2014).

**Lipid profiles, leptin, serum ALP, SGOT and, SGPT measurement**

Total cholesterol (TC), triglyceride (TG), LDL-cholesterol (LDL-c), and HDL-cholesterol (HDL-c) levels and, serum activity of alkaline phosphatase (ALP), serum glutamic-oxaloacetic transaminase (SGOT) and serum glutamic pyruvic transaminase (SGPT) were analyzed by using commercial kits (Pars Azmoon, Iran) and auto-analyzer method. The concentration of very low density lipoprotein cholesterol (VLDL–c) was calculated using the Norbert formula, which equals to one fifth of TG level (Mousavi et al., 2012 ▶). The atherogenic index (AI = Log (TG/HDL-c)) is defined as the zone of atherogenic risk (Rafieian-Kopaei et al., 2014). Serum lLeptin level was evaluated by an ELISA kit (Labor Diagnostika Nord GmbH, Germany) with low-end sensitivities of 0.5 ng/ml, intra and inter-assay CV of 4.3 and 5.8 %, respectively.

**Statistical analysis**

Data were expressed as mean ± standard error of mean (SEM) and analyzed by SPSS using one-way analysis of variance (ANOVA) followed by least significant difference (LSD) test. Statistically significant was considered at p<0.05.

## Results

Effect of *A. lappa* on body weight

**Figure 1:**
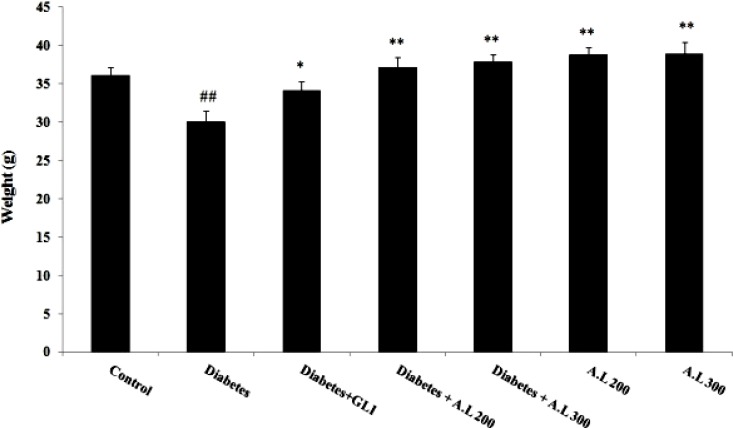
Effect of A. lappa root’s extract on the body weight. Data are expressed as mean ± SEM. *p<0.05 and **p<0.01 vs diabetes group; ##p<0.01 vs control group (n=10 for each group). GLI= Glibenclamide and A.L= A. lappa.

As demonstrated in Figure 1, type 2 diabetes induced a significant decrease in final body weight as compared to control group (p<0.01). Administration of *A. lappa* root extract improved this body weight reduction in type 2 diabetic and healthy mice (p<0.01).

**Table 1:** Effect of *A. lappa* root’s extract on insulin and, fasting blood glucose levels, HOMA-IR, HOMA–β, QUICKI, and DI

	**Glucose** **(mg/dl)**	**I** **nsulin** **(µIU/mL)**	**HOMA-IR**	**HOMA–β**	**QUICKI**	**DI**
**Control**	91.71±4.64	18.92±3.73	4.28±0.78	236.51±47.50	0.31±0.01	3.81±0.68
**Dia**	210.28±11.47###	11.21±1.36#	5.81±0.54	27.38±6.52###	0.30±0.01	1.89±0.34#
**Dia+GLI**	99.16±6.31***	15.50±1.76	3.79±0.55*	154.22±32.91***	0.31±0.005	3.78±0.76*
**Dia** **+A.L200**	109.40±6.55***	19.89±5.37*	5.37±0.91	154.78±29.88***	0.30±0.01	3.09±0.81*
**Dia** **+A.L300**	114.41±10.52***	14.29±2.50	4.03±0.61*	100.28±25.31***#	0.31±0.01	3.38±0.72*
**A.L200**	90.71±4.81***	20.41±2.92*	4.56±0.56	266.67±54.13***	0.31±0.01	3.74±0.85*
**A.L300**	87.85±6.28***	21.46±3.27**	4.65±0.68	311.01±71.40***	0.30±0.01	3.77±0.77*

Data are expressed as mean ± SEM.

* p<0.05,

** p<0.01 and,

*** p<0.001 vs diabetes group;

# p<0.05 and,

### p<0.001 vs control group (n=10). Dia= Diabetic, GLI= Glibenclamide, A.L= *A. lappa*.

**Effect of** ***A. lappa*** **root’s extract on fasting blood glucose and insulin levels**

As shown in Table 1, blood glucose level increased in type 2 diabetes group when compared to other groups (p<0.001). Insulin concentration in diabetic mice decreased compared to control group (p<0.05). Furthermore, *A.* *lappa* 200 mg/kg treated diabetic mice revealed a significant increase in insulin level as compared to type 2 diabetes group (p<0.05). Daily administration of hydro-alcoholic extract of *A.* *lappa* root extract 200 (p<0.05) and 300 mg/kg (p<0.01) revealed a remarkable increase in insulin levels in normal animals when compared to diabetic mice. HOMA-IR as an insulin resistance index, showed a tendency to increase after induction of type 2 diabetes and, this parameter indicated a significant decrease in glibenclamide and *A.* *lappa* 300 mg/kg treated diabetic mice compared to type 2 diabetes group (p<0.05). Beta cell function index (HOMA-β) decreased significantly in type 2 diabetes group as compared to other groups (p<0.001) and the same effect was observed in type 2 diabetes + *A.* *lappa* 300 mg/kg group when compared to control (p<0.05). The insulin sensitivity and insulin secretion, known as the disposition index (DI), was impaired after induction of type 2 diabetes and this parameter showed a significant decrease in diabetic mice as compared to other groups (p<0.05). Ultimately, there was no significant difference in QUICKI as quantitative insulin sensitivity check index among groups.

**Effect of** ***A. lappa*** **on lipid profile**

NA-STZ-induced type 2 diabetes increased AI (p<0.001), serum levels of VLDL, TG (p<0.01), and cholesterol (p<0.05) and decreased HDL level (p<0.01) as compared to control group (Table 2). Treatment of diabetic animals with *A.* *lappa* root extract 200 mg/kg, reduced AI (p<0.01), TG and VLDL (p<0.01) levels and the dose of 300 mg/kg decreased AI (p<0.001), TG and VLDL (p<0.05) levels and increased HDL level (p<0.05) as compared to the diabetic group. Furthermore, extract administration to the intact mice significantly decreased TG and VLDL levels (p<0.01) and AI (p<0.001) at both doses. The levels of TC and LDL were reduced following 4 weeks of treatment with 200 mg/kg (p<0.05) and 300 mg/kg (p<0.01) doses of extract when compared to diabetic groups. Also, serum HDL level increased in mice treated with 200 and 300 mg/kg of extract compared to diabetic group (p<0.01 and p<0.001, respectively). Expect AI, there was no significant difference in lipid profile in glibenclamide-treated diabetic mice in comparison with the diabetic group.

**Effect of** ***A. lappa*** **on serum SGPT, SGOT, ALP and leptin levels**

As shown in Table 3, after induction of type 2 diabetes by NA-STZ, serum ALP, SGPT and SGOT levels increased and serum leptin level decreased compared to control group and these variations were significant for SGPT (p<0.05), ALP and leptin (p<0.01). ALP levels showed a significant decrease in diabetic mice treated with glibenclamide and both doses of the extract (p<0.05) and intact mice treated with the extract (p<0.01) when compared to the diabetic group. *A. lappa* extract administration to intact mice, significantly decreased serum SGPT level at both doses and SGOT level at the dose of 300 mg/kg (p<0.05). Both doses of *A.* *lappa* root extract in healthy and the dose of 300 mg/kg in diabetic mice caused a significant elevation of serum leptin levels compared to diabetic group (p<0.01).

**Table 2.** Effect of *A. lappa* root’s extract on lipid profile (mg/dl) and AI.

	**Cholesterol (mg/dl)**	**TG** **(mg/dl)**	**HDL** **(mg/dl)**	**LDL** **(mg/dl)**	**VLDL ** **(mg/dl)**	**AI**	
**Control**	62.02±6.58	94.71±7.68	43.57±4.21	19.28±2.74	18.94±1.53	0.34±0.03	
**Dia**	84.28±7.43#	128.27±8.97##	23.71±2.67##	32.71±4.67	25.65±1.79##	0.74±0.07###	
**Dia+GLI**	73.16±6.03	118.32±5.85	34.33±3.48	27.66±3.96	23.66±1.17	0.54±0.04*	
**Dia+A.L200**	72.61±6.41	104.62±7.30*	36.4±6.32	24.02±4.31	20.92±1.46*	0.48±0.08**	
**Dia+A.L300**	68.60±6.56	98.23±10.93*	41.21±4.09*	21.41±2.13	19.64±2.18*	0.37±0.05***	
**A.L200**	59.57±6.33*	89.57±6.89**	45.28±6.02**	17.92±1.64*	17.91±1.37**	0.31±0.06***	
**A.L300**	55.71±5.97**	88.71±7.26**	47.85±5.09***	15.42±1.91**	17.74±1.45**	0.27±0.03***

Data are expressed as mean ± SEM.

* p<0.05,

** p<0.01 and,

*** p<0.001 vs diabetes group;

# p<0.05,

## p<0.01 and,

### p<0.001 vs control group (n=10 for each group). Dia= Diabetic, GLI= Glibenclamide, A.L= *A. lappa*.

**Table 3:** Effects of *A. lappa* root’s extract on leptin (ng/dl), SGOT, SGPT and, ALP levels

	**Leptin level** **(U/L)**	**SGPT** **(U/L)**	**SGOT** **(U/L)**	**ALP** **(U/L)**
**Control**	2.19 ± 0.16	60.42 ± 5.55	96.71 ± 8.82	95.28 ± 8.52
**Dia**	1.32 ± 0.18##	128.27 ± 8.97##	23.71 ± 2.67##	127.14 ± 9.26##
**Dia+GLI**	1.91 ± 0.19	54.33 ± 5.63	86.52 ± 9.43	98.83 ± 6.72*
**Dia+A.L200**	1.96 ± 0.21	52.2 ± 4.71	84.24 ± 6.52	101.41 ± 8.17*
**Dia+A.L300**	2.26 ± 0.19**	51.23 ± 4.61	80.81 ± 9.68	99.62 ± 7.96*
**A.L200mg/kg**	2.24 ± 0.24**	45.42 ± 4.76*	75.14 ± 6.54	94.42 ± 7.36**
**A.L300mg/kg**	2.37 ± 0.28**	45.14 ± 5.31*	73.42 ± 8.08*	91.85 ± 9.63**

Data are expressed as mean ± SEM.

* p<0.05 and,

** p<0.01 vs diabetes group;

# p<0.05,

## p<0.01 and,

### p<0.001 vs control group (n=10 for each group). Dia= Diabetic, GLI= Glibenclamide, A.L= *A. lappa*.

## Discussion

Type 2 diabetes mellitus as a heterogeneous metabolic syndrome that leads to insulin secretion or insulin resistance defects (Roghani et al., 2013 ▶). In this study, NA and STZ were applied to induce type 2 diabetes in mice. STZ as a diabetogenic agent, was used to induce type 1 diabetes by induction of oxidative stress in beta cells (Raza and John, 2012 ▶). Also, NA protects beta cells from STZ-induced damage through its antioxidant property and induces type 2 diabetes (Kamat and Devasagayam, 1999 ▶). 

As it has been demonstrated in one study (Ghamarian et al., 2012 ▶), the present research also indicates that serum insulin level significantly reduced in the diabetic group. Hence, it can be attributed to the ability of STZ in generation of ROS and insulin production impairment by beta cells (Raza et al., 2011 ▶). In this study, insulin levels in diabetic mice imply that some healthy beta cells remained to produce some insulin. Also, glucose level was increased in diabetic mice. One of anti-hyperglycemic mechanisms is increasing insulin release and decreasing glucose absorption from the intestine (Ovalle-Magallanes et al., 2015 ▶). The results of this investigation showed that *A. lappa* root extract reduced blood glucose and elevated serum level of insulin in treated diabetic animals. So, it can be suggested that the possible hypoglycemic mechanism of *A. lappa* root extract is restoration of pancreatic tissue function and improvement of insulin production or decreasing the intestinal glucose absorption.

Diabetogenic agents such as STZ can cause oxidative damage to beta cells. According to a report, flavonoids are able to protect the beta cells function through their free radical scavenging properties in islets of Langerhans (Patel et al., 2012 ▶). The important compounds of *A.* *lappa* root extract are flavonoids, alkaloids and saponins (Al-Shammaa et al., 2013 ▶). Several investigators have noted that flavonoids are strong bioactive antioxidant and antidiabetic agents and the alkaloid content of plants could modulate insulin secretion. Also, saponins have blood glucose lowering effect (Patel et al., 2012 ▶). So, *A. lappa* roots extract activity to improve beta cells function, may be due to the presence of these components. The insulin-related biomarkers, including QUICKI, HOMA-IR, HOMA-β, and DI were calculated to reveal the health of insulin-producing cells and function of insulin in type 2 diabetes. HOMA-IR model has demonstrated to be a strong clinical and epidemiological tool for assessment of insulin resistance, but QUICKI and HOMA-β are negatively correlated with HOMA-IR (Patel et al., 2012 ▶). Hence, present results indicate that *A. lappa* roots extract have improvement effects in type 2 diabetes complications through enhancement of beta cell function, induction of insulin sensitivity and insulin secretion, and reduction of insulin resistance index.

Type 2 diabetes affects glucose and lipid metabolism. Insulin deficiency diminishes lipoprotein lipase (LPL) activity. Free fatty acid influx stimulates hepatic triglyceride synthesis and increases the production of LDL. Also, VLDL, TG and TC levels increase while HDL decreases in uncontrolled type 2 diabetes condition (Indradevi et al., 2012 ▶), which was in agreement with this study. Also, in agreement with the present study, Cao et al. revealed that *A. lappa* roots ethanolic extract improved hyperlipidemic conditions in type 1 diabetic rats near to the normal levels. The probable mechanisms of this effect on serum lipid profile improvement are inhibition of HMG-CoA reductase and cholesterol absorption from the intestines due to formation of complexes with components such as glycosides and saponins (El-Soud et al., 2007 ▶). Hence, the hypolipidemic effects of *A.* *lappa* extract may be mediated through the above-motioned mechanisms and due to the presence of its compounds. Also, lipogenesis is reduced during type 2 diabetes due to underutilization of glucose (El-Soud et al., 2007 ▶). Also, alkaloids of *A.* *lappa* have been shown to stimulate hepatic lipogenic enzymes and decrease TG levels (Raju et al., 2001 ▶). Moreover, in the present study, the atherogenic index was lowered in diabetic rats and increased following the treatment with *A.* *lappa*. Hence, this finding may suggest a negative relationship between administration of *A.* *lappa* and risk of atherothrombotic disease.

STZ-induced diabetes was associated with weight loss through muscle wasting and loss of tissue protein (Rangachari and Savarimuthu, 2012 ▶). Insulin stimulates the influx of amino acids into the skeletal muscle to increase protein synthesis (Long et al., 2011 ▶). So, weight reduction occurs as a result of the progressive decline of insulin action in diabetes. Our findings about body weight are consistent with the findings of other studies (Ghamarian et al., 2012 ▶) that showed mean body weight reduction in diabetic cases. In the present study, treatment of diabetic mice with *A.* *lappa* root extract was effective against excess body weight loss in a concentration-dependent manner.

Our results regarding lipid profiles after oral administration of *A.* *lappa* extract indicate lipid lowering effect of the extract. So, the weight gain effect may be attributed to all other mechanism excluding lipid metabolism pathway. One plausible mechanism against body weight loss is amelioration of serum insulin level in diabetic mice. Furthermore, this study showed that doses of burdock root extract not only have a protective effect on weight loss, but also result in gain weight in the control group (healthy mice).

The main tissue for insulin-dependent glucose uptake is liver which plays an essential role in glucose and lipid homeostasis (Chu et al., 2014 ▶). Induction of diabetes cause to hepatocellular damage and characterized by high serum levels of ALP and SGPT (Singh et al., 2009 ▶; Kazemian Mansur Abad et al., 2015 ▶). 

One study indicated that *A. lappa* decreased SGOT and SGPT elevations in carbon tetrachloride-induced hepatotoxicity in mice (Lin et al., 2000 ▶). In the current study, elevations of serum SGPT and ALP levels were observed when compared to control group. Hence, it can be suggested that the reversal effect of *A. lappa* extract on ALP and SGPT levels occurred through prevention of hepatocellular damage induced by NA-STZ type 2 diabetes conditions. 

Administration of *A.* *lappa* root extract and glibenclamide elevated serum leptin level, which was decreased in the diabetic group. Leptin reduction has been reported in diabetic animals (Andaloussi et al., 2011 ▶) and is probably induced through the impairment of glucose uptake and adipose tissue metabolism. Insulin helps glucose uptake and oxidation in adipocytes and increases serum level of leptin. The present study revealed that *A.* *lappa* root extract increased serum insulin level in diabetic mice, so the effect of this extract on leptin level might be correlated with its beneficial effect on insulin release from beta cells (Zhang and Benny, 2000 ▶).

In conclusion, present results suggest that *A.* *lappa* root extract has hypoglycemic effect and this extract can ameliorate lipid profiles as well as hepatic enzyme levels in diabetic mice. Therefore, this plant extract can be useful in treating type 2 diabetes mellitus as mentioned in Iranian traditional medicine. However, future investigations are necessary to understand the exact mechanisms of antidiabetic effects of *A.* *lappa* root extract. 
